# Contraceptive uptake in post abortion care—Secondary outcomes from a randomised controlled trial, Kisumu, Kenya

**DOI:** 10.1371/journal.pone.0201214

**Published:** 2018-08-10

**Authors:** Marlene Makenzius, Elisabeth Faxelid, Kristina Gemzell-Danielsson, Theresa M. A. Odero, Marie Klingberg-Allvin, Monica Oguttu

**Affiliations:** 1 Department of Public Health Sciences, and Global Health (IHCAR), Karolinska institutet, Stockholm, Sweden; 2 Deptartment of Women´s and Children´s Health, Karolinska institutet, Stockholm, Sweden; 3 Karolinska University Hospital, Stockholm, Sweden; 4 College of Health Sciences, School of Nursing Sciences, University of Nairobi, Nairobi, Kenya; 5 School of Education, Health and Social studies, Dalarna University, Falun, Sweden; 6 Kisumu Medical Education Trust–KMET, Kisumu, Kenya; National Institute of Health, ITALY

## Abstract

**Aim:**

The aim was to explore contraceptive uptake, associated factors and satisfaction among post abortion-care (PAC) seeking women in Kenya. Due to unsafe abortions, almost 120 000 Kenyan women received PAC in 2012, and of these women, 70% did not use contraception before pregnancy.

**Methods:**

This study was nested in a larger randomised controlled trial, where 859 women sought PAC at two public hospitals in Kisumu, in June 2013–May 2016. The women were randomly assigned to a midwife or a physician for PAC, including contraceptive counselling, and followed up at 7–10 days and three months. Associated factors for contraceptive uptake were analysed with binary logistic regression, and contraceptive method choice, adherence and satisfaction level were examined by descriptive statistics, using IBM SPSS Statistics for Windows, Version 22.0.

**Results:**

Out of the 810 PAC-seeking women, 76% (n = 609) accepted the use of contraception. Age groups of 21–25 (OR: 2.35; p < 0.029) and 26–30 (OR: 2.22; p < 0.038), and previous experience of 1–2 gravidities (OR 1.939; p = 0.018) were independent factors associated with the up-take. Methods used: injections 39% (n = 236); pills 27% (n = 166); condoms 25% (n = 151); implant 7% (n = 45) and intrauterine device (IUD) 1% (n = 8). At 3-month follow-up of the women (470/609; 77%), 354 (75%) women still used contraception, and most (n = 332; 94%) were satisfied with the method. Reasons for discontinuation were side-effects (n = 44; 39%), partner refusal (n = 27; 24%), planned pregnancy (n = 27; 24%) and lack of resupplies (n = 15; 13%).

**Conclusions:**

PAC-seeking women seem highly motivated to use contraceptives, yet a quarter decline the use, and at 3-month follow-up a further quarter among the users had discontinued. Implant, IUD and permanent method are rarely used. Strategies to improve contraceptive counselling, particularly to adolescent girls, and to increase access to a wide range of methods, as well as provider training and supervision may help to improve contraceptive acceptance and compliance among PAC-seeking women in Kisumu, Kenya.

## Introduction

Many regions of the world have high numbers of unwanted pregnancies and unmet contraceptive needs [1–5]. An estimated half of sexually active women of reproductive age (15–49 years) in low-income countries or 818 million women, want to avoid pregnancy [6]. Among these women, about 17%, or 140 million, do not use any contraception, while 9%, or 75 million, use less-effective traditional methods. Altogether, 215 million women are said to have unmet needs for modern contraception [6]. These women are potential users of contraception.

Access to contraception supports people’s rights to determine the number and spacing of children and reduces the need for induced abortions, especially unsafe abortions [1, 6, 7]. The case-fatality rate is estimated to be 460 deaths per 100,000 unsafe abortions on the African continent, compared to 30 deaths per 100,000 in high-income countries [8]. These deaths are almost entirely preventable, and contraception is a primary preventive strategy, with evident beneficial effects beyond maternal health: it increases the economic wellbeing of individuals, families, communities and nations and promotes environmental sustainability [1, 6, 8].

Kenya’s total fertility rate is 3.9 children during a woman’s lifetime [9]. More than half of adolescent girls would like to delay childbearing, and about half desire to have three or fewer children [9]. However, less than 20% of adolescent girls use any contraceptive method (2008–2009), and less than 15% use long-acting reversible contraceptive (LARC) methods, such as implants, intrauterine devices (IUD) and injections [9]. Although use of LARC methods increased slightly from 10% in 1998 to 14% in 2008–2009 [9], only 12%–17% of Kenya’s poorest and uneducated married women have ever used LARC, compared with 48%–52% of the wealthiest and most educated married women [9].

An estimated 465,000 induced abortions, mostly using unsafe procedures were performed in Kenya in 2012, a rate of 48 per 1,000 women of reproductive age [9, 10]. Unsafe abortion is defined as a procedure for terminating an unwanted pregnancy either by persons lacking the necessary skills or in an environment lacking minimal medical standards or both [11]. Nearly 120,000 Kenyan women received post abortion care (PAC) in health care facilities for complications resulting from unsafe abortions in 2012 [4]. Of these women, 70% were not using a contraceptive method before becoming pregnant [4]. Efforts to assist with contraceptive method selection and to improve the content of contraceptive counselling by providers have the potential to increase contraceptive use in urban Kenya [12].

PAC is widely recognized as a critical practice to address complications related to miscarriage and incomplete abortion and reduce repeat abortions by the provision of contraception services [5, 13–17]. Most contraceptive methods can, and are indeed recommended to be initiated immediately after an abortion because ovulation can resume within 10 days of abortion [18, 19]. Although, to initiate contraception, is challenging, both from a client and a provider perspective. Widespread reasons for non-use, are particularly infrequent sex and concerns regarding side effects or health risks [20]. The providers requires medical knowledge and training in counselling skills, and a variety of contraceptive methods needs to be available during PAC [12, 18, 21, 22]. The aim of this study was to investigate contraceptive up-take and associated factors, among women who received PAC from midwives and physicians in Kisumu, Kenya.

## Methods

This paper is based on analyses of secondary outcomes from a randomised controlled trial with the aim to assess the effectiveness of midwives administering misoprostol to women with incomplete abortion seeking PAC, compared with physicians [16]. The project was conducted in Kisumu County in the Nyanza/Western region, at Jaramogi Oginga Odinga Teaching and Referral Hospital and Kisumu County Hospital. The hospital are situated in Kisumu town, Kenya’s third-largest city with an estimated population of 500,000. Regional variations in the incidence of abortion exist in Kenya, and the Nyanza/Western region has among the highest rates: 63 induced abortions per 1,000 reproductive-age women, compared with 48 per 1,000 nationally [9]. In this region, only 37% of women have ever used a contraceptive method compared to 46% nationally [9]. The project was implemented in the gynaecological ward of the two facilities’ obstetrics and gynaecology departments from 1 June 2013 through 31 August 2016 [16]. Together, the two facilities admit approximately 26 women with incomplete abortions per month. The midwives (n = 19) and physicians (n = 18) included in the project underwent standardised training in post abortion care including contraceptive counselling [13, 19, 23].

Counselling space was provided in a secluded room and to securely store the large number of protocol records. The study was designed and coordinated by researchers at the Department of Public Health Sciences, Global Health (IHCAR), and Department of Women’s and Children’s Health, Karolinska Institutet, Stockholm, Sweden; Kisumu Medical Education Trust; University of Nairobi, Kenya; Moi University, Eldoret, Kenya; and Jaramogi Oginga Odinga Teaching and Referral Hospital and Kisumu County Hospital, Kisumu, Kenya. The research questions and outcome measures were developed by researchers and health care providers experienced in PAC at the studied facilities[16].

The Jaramogi Oginga Odinga Teaching and Referral Hospital Ethical Review Committee (diary number ERC42/13) and the Swedish regional Ethics Committee in Stockholm (2013/902-31/1) gave ethical approval for the study. All the patients provided written, informed consent and could withdraw from the study at any time without consequences [16].

### Inclusion and exclusion criteria

The inclusion criteria were women presenting with vaginal bleeding in the first trimester of pregnancy and diagnosed to have incomplete abortion, and the exclusion criteria were omen with unstable hemodynamic status and shock, signs of sepsis [16].

### Randomisation and masking

Eligible women who consented to participate were randomly assigned to a midwife (intervention) or standard care with a physician (control) for diagnosis and treatment [16]. Data management was organised locally at the coordinating department at Kisumu Medical Education Trust, Kisumu. The providers received support and guidance from the study coordinator throughout the study period. The study was not masked to either the study participants or the providers. The trial is registered at ClinicalTrials.gov, number NCT01865136.

### Procedures

From 1 June 2013 to 31 May 2016, women seeking PAC were recruited (n = 859), by midwives and physicians involved in PAC at two facilities in Kisumu. The health care providers took part in a 5-day theoretical and practical training program which followed the Kenyan and the World Health Organization’s standardised PAC training module and medical eligibility criteria for contraceptive use [13, 19, 23]. On-going, facility-based, continuing medical education was provided for all staff, and new staff, throughout the project period. This training included interview techniques, diagnosis of incomplete abortions, treatment with misoprostol and MVA and post abortion contraceptive counselling and provision [13, 19].

Four midwives per facility were trained as research assistants and were responsible for screening and enrolment of participants. The midwives role was also to monitor the supply stock (misoprostol and contraceptives, such as condoms, combined-pills, depot medroxyprogesterone acetate injections, hormonal implants and IUDs). The research assistants conducted the follow-up visits, including assessing abortion status and the contraceptive uptake.

Women admitted with signs of incomplete abortion were screened based on self-reported last menstrual period, pregnancy test, symptoms and clinical assessments. Eligible women who consented to participate were randomly assigned to the midwife or the physician group for the clinical assessment [16]. Each participant was given a single dose of 600 μg misoprostol orally, and before discharge all the women were offered contraceptive counselling, provided with a contraceptive method to be started if they desired it. IUD and Implant were charge with a fee of US$5, while pills (one month), 10–20 condoms, and injection (lasting for three month) was provided free of charge. They were scheduled for follow-up appointments at 7–10 days and three months. At three months they were also offered to refill their choice of contraceptive method if needed for a fee of US$1 (pills, 10–20 condoms and injection).

The women were offered reimbursement for travel costs as an incentive to attend the follow-up appointment. All the women received an SMS reminder two days before their scheduled appointments. Women who did not show up were contacted by a research assistant using a cell phone provided by the project, and the women was given new appointments and encouraged to attend. No serious adverse events were recorded.

### Outcomes

The outcome for this paper was the contraceptive uptake among the PAC-seeking women who were followed up (n = 810/859) as assessed by background characteristics (Table 1) and six questions:

- Did you receive contraceptive counselling? (7–10-day follow-up)
- Did you accept to start a contraceptive method? (7–10-day follow-up)
- What method did you chose to start? (7–10 day follow-up)
- Are you still using the method? (3-month follow-up)
- What is your satisfaction level with the chosen method? (3-month follow-up)
- What is your reason for interrupting the method? (3-month follow-up)

**Table 1:** Participants’ characteristics (n = 810) by provider.

Characteristics^a^	Midwife group n (%)	Physician group n (%)	Total sample^b^ n (%)
**Age (years)**			
N	409 (50.5%)	401 (49.5%)	810
Mean (SD)	25.28 (5.81)	24.85 (5.46)	25.07 (5.64)
Range	14–45	14–41	14–45
**Marital status**			
N	409	401	810
Married or cohabiting	277 (67.7%)	277(69.1%)	554 (68.4%)
Single/divorced/separated/widowed	132 (32.3%)	124 (30.9%)	256 (31.6%)
**Religion**			
N	409	401	810
Christian	404 (98.8%)	392(97.8%)	796 (98.3%)
Muslim	5 (1.2%)	9 (2.2%)	14 (1.7%)
**Education**			
N	409	401	810
None	5 (1.2%)	2 (0.5%)	7 (0.9%)
Primary grades 1–8	108 (26.4%)	127 (31.6%)	235 (29%)
Secondary education	198 (48.4%)	169 (42.1%)	367 (45.3%)
Tertiary education	98 (24%)	103 (25.7%)	201 (24.8%)
**Occupation**			
n	407	398	805
Unemployed	195 (47.9%)	184 (46.2%)	379 (47.1%)
Formal employment/self-employment	212 (52.1%)	214 (53.7%)	426 (52.9%)
**Gestational age based on Last menstrual Period (weeks)**			
N	409	401	810
Mean (SD)	9.4 (2.2)	9.7 (2.1)	9.6 (2.2)
Range	1–12	3–12	1–12
**Previous gravidity**			
N	409	401	810
Mean (SD)	1.76 (1.84)	1.82 (1.58)	1.79 (1.72)
Range	0–12	0–8	0–12
**Parity (live births)**			
n	407	401	808
Mean (SD)	1.09 (1.43)	1.13 (1.27)	1.1 (1.35)
Range	0–10	0–7	0–10

^a^ Data are n (%) unless otherwise stated.

^b^ The internal drop-out had a range of 0–8 (0%–2.2%).

### Statistical analyses

The sample size calculation was based on the main outcome, assuming that the rate of incomplete abortions was 4% [16]. The participants’ background characteristics are presented with descriptive statistics (M, SD and range). A nonlinear correlation between contraceptive acceptance, and age and previous gravidities. Age was therefore grouped into: 14–20; 21–25; 26–30; 31–35 and 35–45 years. Women with previous gravidity experience was grouped into nulligravidity, 1–2 and 3–12 gravidities.

To determine the factors associated with women’s acceptance of starting to use a contraceptive method and the adherence at three months, Pearson’s chi-squared test was used, and the significance level was set at < 0.05. The same method was used to investigate possible differences between the two provider groups (midwife and physicians). In addition, a binary logistic regression model was used to identify the factors associated with the dependent variable *acceptance to start a contraceptive method*. The variables in the bivariate analysis that had a *p value* < 0.05 were used as explanatory variables (age, marital status and parity). To adjust for possible interactions between the variables, each variable was controlled against the others, and correlations of less than 0.70 were used. The associations were presented as odds ratios (OR) with a 95% confidence interval (CI). IBM SPSS Statistics for Windows, Version 22.0 was used for all the analyses. All the authors had full access to the data, and the first author regularly reported the preliminary results to the entire research team. The corresponding author had final responsibility for the decision to submit the manuscript for publication.

## Results

Out of the 859 women, a total of 49 (6%) women were lost to follow-up (24 in the midwife group and 25 in the physician group). Finally, a total of 810 women, 409 in the midwife group and 401 in the physician group, received PAC due to first-trimester incomplete abortion and were included in the per-protocol analysis. Table 1 shows the background characteristics of the 810 participating women, similar distributed between the midwife and the physician groups. About half of the women were accompanied by their partners to the clinic (n = 436; 54%).

### Acceptance of post abortion contraception

Among 810 women who were followed-up at 7–10 days, most (n = 789; 97%) reported that they had received contraceptive counselling at the first visit, and 609 women (75%) had accepted starting the contraceptive method of their choice. There were no differences in the acceptance rate between the midwife and the physician groups (*p* = 0.249).

Table 2 shows the distribution of the contraceptive methods chosen, among the 609 women: injections n = 236 (39%); pills n = 166 (27%); condoms n = 151 (25%); implants n = 45/614 (7%) and IUDs n = 8 (1%). The distributions were similar for the midwives and the physicians groups. One woman chose a permanent method but at follow-up she was awaiting the appointment, but ultimately she did not undergo the surgical procedure.

**Table 2:** Contraception acceptance among women seeking post abortion care.

Contraceptive Methods^a^ (Duration of supply)	Women who accepted the use (n = 609/810) n (%)			*P value*^b^
	Midwife group n = 301	Physician^a^ group n = 308	Total sample n = 614	
Injection (3 months)	118 (39.2)	118 (38.6)	236 (38.9)	0.871
Pills (1 month)	81 (26.9)	85 (27.8)	166 (27.3)	0.811
Condoms (n = 10–20)	75 (24.9)	76 (24.8)	151 (24.9)	0.982
Implant (3 years)	24 (8)	21 (6.9)	45 (7.4)	0.602
IUD (5 years)	3 (1)	5 (1.6)	8 (1.3)	-
Permanent method	-	1 (0.2)	1 (0.2)	-

^a^ Contraceptive method was not reported for two women.

^b^ Pearson’s χ 2 test was used and the significance level was set at < 0.05.

Table 3 shows bivariate analysis of the women’s (n = 810) acceptance of PAC contraception and the associated factors. The associated factors for acceptance of starting a contraceptive method of women’s choice were to be married or cohabiting (*p* = 0.021), previous gravidity experience (*p* = < 0.01), parity (*p* = 0.01) and to be accompanied by partner at the clinic (*p* = 0.02). A nonlinear correlation between age and contraceptive acceptance was identified, showing the largest proportion of acceptance among women aged 21–25 years (n = 236; 79.2%) and 26–30 years (n = 165; 80.9%), but falling off in age groups of 31–35 years (n = 53; 70.7%) and 35–45 years (n = 32; 69.6%). The proportion of acceptance was lowest in the age group 14–20 years (n = 115; 68%). With regard to previous gravidity experience, the largest proportions of acceptance were among women with previous experience of 1–2 gravidities (n = 273; 78%) and 3–12 gravidities (n = 189; 80%), compare to nulligravidity (n = 152; 68%) (*p* = 0.007).

**Table 3:** Post abortion contraception. Women’s acceptance of starting a contraceptive method and associated factors (n = 810 women).

Associated factors	Women (n = 614/810) who accepted starting a contraceptive method n (%)	*P value*^a^
Married or cohabiting	433 (78.2)	0.021
Reference: Single/divorced/separated/ widowed	181 (70.7)	
Secondary and tertiary education	429 (75.5)	0.780
Reference: None or primary-school grades 1–8	185 (76.4)	
Employed	326 (76.5)	0.599
Reference: Unemployed	284 (74.9)	
Nulligravidity	152 (68.2)	0.002
Reference: Previous gravidity 1–12	462 (78.7)	
Nulliparous	253 (71.1)	0.006
Reference: Parity 1–10	359 (79.4)	
Accompanied by partner to the clinic	343 (78.7)	0.032
Reference: Not accompanied	264 (72.1)	

^a^ Pearson’s χ 2 test was used and the significance level was set at < 0.05.

A binary logistic regression (n = 792/810 women) was used to assess the independent factors associated with the dependent variable the *acceptance of starting a contraceptive method*, presented as OR with a 95% CI (Table 4). The variables used in the model were associated factors with a *p value* < 5% in Table 3. The high correlation between the experience of previous gravidity and previous live birth, resulted in the use of pregnancy experience in the model, only. The predictor for acceptance of PAC contraception were to be in the age groups of 21–25 (OR 2.347; *p* = 0.029) and 26–30 years (OR 2.22; *p* = 0.038) and to have experienced 1–2 previous gravidities (OR 1.932; *p* = 0.018). The Hosmer and Lemeshow Test showed a non-significant *chi2* (*p* = 0.947) and indicated that the model fits the data well.

**Table 4:** Binary logistic regression model. Predictors of starting a contraceptive method among women (n = 792) obtaining post abortion care.

Predictors	Association with acceptance of starting contraceptive methods		
	Women (n = 792)		
	OR^a^	CI 95%	*p value*
Age 14–20 years	1.576	0.692–3.590	0.279
Reference: 36–45 years			
Age 21–25 years	2.347	1.090–5.054	0.029
Reference: 36–45 years			
Age 26–30 years	2.220	1.044–4.722	0.038
Reference: 36–45 years			
Age 31–35 years	1.141	0.507–2.568	0.749
Reference: 36–45 years			
Married or cohabiting	0.865	0.567–1.321	0.503
Reference: sngle/divorced/separated/widowed			
Nulligravidity	1.483	0.982–2.241	0.061
Reference: multigravidity 3–10			
Gravidity 1–2	1.932	1.118–3.337	0.018
Reference: multigravidity 3–10			
Partner accompanied to the clinic	0.825	0.575–1185	0.298
Reference: Not accompanied			

^a^Binary logistic regression model, presented as odds ratio (OR) with 95% confidence interval (CI)

### Follow-up at three months

Among the 609 women who started contraceptive methods, 470 (77%) were followed up at three months. At that time, 354/470 (75%) were still using the method, and 94% (n = 332/470) of these women were highly satisfied or satisfied with the method. Nine women were not satisfied, and 13 women wanted to stop using their method (among them 11 were using hormonal injection), and one women were never provided the requested permanent method.

Among the 115 women (25%) who had interrupted continuation of their contraception were distributed by following methods: pills = 35/126 (28%); injection = 33/188 (18%); condoms = 31/112 (28%); implant = 9/32 (28%), and IUD = 5/6 (83%). The interruption rate was similar for both the midwife group (n = 57/230; 25%) and the physician group (n = 59/240; 25%). The contraceptive method chosen was not reported for two women.

The most common reasons for interruption were side-effects, mainly bleeding problems (n = 44; 39%), partner refusal (n = 27; 24%), desire for pregnancy (n = 27; 24%) and lack of resupplies (n = 15; 13%). None of the associated background factors (Table 3) had a significant relationship (*p* < 0.05) with adherence to the chosen method at the 3-month follow-up.

## Discussion

The aim of this study was to investigate contraception up-take, associated factors and satisfaction among women who received post abortion contraceptive counselling from midwives and physicians in a low-resource setting in Kenya. The results show high uptake and satisfaction of post abortion contraception, in particular among women in the age of 21–30 years and among women with previous gravidity experience. However, a quarter of the women declined to start a contraceptive method, and a further quarter of the women had interrupted the use of the method at 3-month follow up. The most common reasons were side-effects such as bleeding problems, partner refusal and desire for a pregnancy, aligning with other studies [7, 20, 24, 25]. No association to background characteristics was detected to the discontinuation.

The most commonly chosen contraceptive method among the PAC-seeking women were injection, followed by pills and condoms, similar to national rates of methods used in Kenya [9]. Although implant and IUD have been proven to have the highest rates of satisfaction and continuation of all reversible contraceptives [26], this could not be proved in this study. Contributing factor were the low use of these methods, and the high discontinuation rate for IUD.

The second most common reason for contraceptive discontinuation was the refusal of male partners to use the method, which was as common as the desire for a new pregnancy. In line with this finding, there was also an association between the woman’s contraceptive acceptance and male partner accompany to the clinic. Improving male involvement in the prevention of unintended pregnancies, which ought to be a shared responsibility, should be encouraged [27, 28]. However, men’s reproductive control of women’s fertility is also an important factor undermining women’s reproductive autonomy [29–31]. Previous research has shown that some men use the strategies of intimidation, threats and actual violence to control women’s fertility with the aim, for example, to promote pregnancy through sabotaging use of a contraceptive method, to force termination of a pregnancy (safe or unsafe) or to interfere with abortion care [30].

Age (21–30 years) and previous pregnancy experience were found to be predictors of acceptance of starting a contraceptive method among PAC-seeking women. The participating women origin from Western Kenya, a poor and low-income region, which may have introduced bias to identify association between contraception and acceptance with regard to socioeconomics (educational level or employment). However, such association is expected as these variables are theoretically connected [32] and also shown in similar studies [33, 34]. Formal schooling adds significant value to innate ability in the form of higher-order cognitive skills crucial to decisions about health [32].

The association between contraception acceptance, and being married (or cohabiting) and to be accompanied by partner to the clinic were eliminated when adjusted for age and gravidity in the regression model (Table 4). This can be explained by findings from other studies from Kenya revealing that religious leaders, family, and health care providers were viewed as reinforcing cultural expectations for married women to have children [33, 35, 36]. A notable finding was the considerably high proportion (32%) of young women (14–20 years) who did not accept contraception, compared to the older age groups. This low approval of contraception among sexually active adolescents is worrying, since statistics shows that more than half of Kenyan adolescent girls want to delay or space childbearing [9]. A possible explanation could be the widespread and well-recognised stigmatising attitudes surrounding young women’s sexuality [21, 36]. Young women are stigmatised for having sex at a young age and before marriage, and the use of contraception may increase such stigma. Studies shows that service providers remain averse to encouraging young people to space or delay pregnancy [21, 35, 36]. Negative attitudes of service providers are based on social-cultural barriers such as religious beliefs and negative gender norms, and misinformation, myths and misconceptions [36].

### Limitations and strengths

The results of this study show that implementation of systematic provision of contraceptive counselling and provision of contraceptives in PAC are feasible and has great potential for future quality development. However, the facilities in this setting were challenged by limited resources, shortages of staffs, skills and inadequate supply chain management. Injection was the most commonly chosen method and may have been caused by heavy workload due to shortage of staffs, as injections are the easiest method to provide. Nevertheless, injections are known to have higher risks for side-effects than other contraceptives, especially IUDs and implants [20]. In this study, pills and depot medroxyprogesterone acetate injections were free of charge, while a US$5 fee was charged for IUDs and implants, possibly one reason why few women chose these methods. In some cases, women who agreed to pay the fee were advised to visit another facility in the hospital area to receive the method from a trained, skilled provider. However, some women were reluctant to visit another facility or provider as the treatment was sensitive, as in Kenya, induced abortion without a medical health reason is considered to be a crime [35, 36]. The referral of women was difficult to avoid due to staff shortage end new providers sometimes entered the study with short notice and were not given timely training to insert implants and IUD.

Add to the problem that sporadic stock-outs of contraceptive methods occurred throughout the study period (supply chain management and financing). The limited capacity to offer a full choice of methods as they did not always have readily assembled IUDs and implant kits, added to the weakness of this study. These obstacles were not identified as an issue that needed to be addressed during the baseline assessment, because all contraceptives were available at that time, and all providers were trained. When these issues were identified later, it was difficult to catch up, solve and prevent them. Moreover, it precluded rigorous statistical analysis on these methods.

In addition, the questionnaire was not designed to measure contraception uptake as a primary outcome as the main outcome was the use of misoprostol to treat incomplete abortion [16]. Nevertheless, the results emphasise the essentials to improve provider training on post abortion contraceptive counselling, in particular to young women, increase the availability of IUDs and implants, but also permanent methods.

### Implications and future research

The high discontinuation rate for the IUD use indicates insufficient continued follow-up and staff training on IUD, and must be further explored and improved. Moreover, the proportion of non-acceptance of a contraceptive method and the proportion of discontinuation points to several barriers to obtain and provide post abortion contraception. Obstacles among PAC-seeking women include side effects, lack of resupplies and partner disapproval of contraceptive use. Barriers at the provider level may reflect lack of skills, denial of certain methods, and disapproval of contraception to adolescents or women who have not yet delivered a child. To develop strategies mitigating these barriers are urgent, such may include provider training and supervision on contraceptive counselling, timing of return to fertility, side-effects and health concerns. In addition, timely informed method-switching needs to be better recognised in PAC to avoid discontinuation and the risk of sub-sequent unintended pregnancies, unsafe abortion and unwanted or mistimed births.

At the facility and policy level further development is need to improve contraceptive services so that women can make informed choices among a wide range of methods, and obtain the chosen method before discharge from the facility. In addition, more research is required to understand underlying reasons for contraceptive use and non-use among young women. Such as better understanding of the impact that socio-cultural stigma, negative gender norms, and male-involvement may have on the woman’s contraceptive acceptance, choices and compliance.

## Conclusions

The findings show that systematic provision of post abortion contraception is feasible in a low-resource setting in a country with restrictive abortion regulations, but need further development. The PAC-seeking women in this study seem highly motivated to use contraceptive methods, and associated factors were to be in the age of 21–30 years and to have had previous gravidity experience. Yet, a quarter decline contraceptive use and at three month follow up an additional quarter had discontinued among the users. Injection was the most common choice, while implant and IUD, were rarely used. None received permanent methods. Moreover, these facilities should place priority on provider training and supervision on a wide range of methods, and to make them affordable to increase women’s access to a full range of contraceptives, comprehensive and accurate information, in particular to adolescent girls.

## Supporting information

S1 File Binary logistic regression: Predictors of starting a contraceptive method among women obtaining post abortion care. (PDF)
