# Supplementary material for: Contraceptive uptake in post abortion care—Secondary outcomes from a randomised controlled trial, Kisumu, Kenya
Source: PLoS One. 2018 Aug 10;13(8):e0201214. doi: 10.1371/journal.pone.0201214 (PMC6086397; doi:10.1371/journal.pone.0201214)
Supplement: S1 File — (PDF) [file pone.0201214.s001.pdf]

Binary Logistic Regression: Predictors of starting a contraceptive method among women (n=792) obtaining post abortion care.

Notes

Output Created		05-DEC-2017 14:36:45
Comments		
Input	Data	\\fohm\public\home_osd\marlene.makenzius\Documents\KENYA POSTDOC\Contraceptives\REVISED SUBM 2\DATAsharing Plosone.sav
	Active Dataset	DataSet1
	Filter	<none>
	Weight	<none>
	Split File	<none>
	N of Rows in Working Data File	810
Missing Value Handling	Definition of Missing	User-defined missing values are treated as missing
Syntax		LOGISTIC REGRESSION VARIABLES ACCEPTCONTR<br>/METHOD=ENTER AGE5 Status2 Preg3 Q3.9<br>/CONTRAST (AGE5)=Indicator<br>/CONTRAST (Status2)=Indicator<br>/CONTRAST (Preg3)=Indicator(1)<br>/CONTRAST (Q3.9)=Indicator<br>/PRINT=GOODFIT CORR CI(95)<br>/CRITERIA=PIN(0.05) POUT(0.10) ITERATE(20) CUT(0.5).
Resources	Processor Time	00:00:00,05
	Elapsed Time	00:00:00,04

Case Processing Summary

Unweighted Cases[a]		N	Percent
Selected Cases	Included in Analysis	784	96,8
	Missing Cases	26	3,2
	Total	810	100,0
Unselected Cases		0	,0
Total		810	100,0

a. If weight is in effect, see classification table for the total number of cases.

Dependent Variable Encoding

Original Value	Internal Value
No	0
YES	1

Categorical Variables Codings

			Parameter coding			
		Frequency	(1)	(2)	(3)	(4)
AGE5	-20	168	1,000	,000	,000	,000
	21-25	293	,000	1,000	,000	,000
	26-30	203	,000	,000	1,000	,000
	31-35	74	,000	,000	,000	1,000
	36-	46	,000	,000	,000	,000
Pregnancies 3 cat	0	219	,000	,000		
	1-2	339	1,000	,000		
	3-12	226	,000	1,000		
Did your partner accompanied you to the PAC clinic?	Yes	427	1,000			
	No	357	,000			
Status dic	Married/co-habiting	537	1,000			
	Single/widowed/devorced/separated	247	,000			

Block 0: Beginning Block

Classification Table[a,b]

	Observed		Predicted		
			ACCEPTCONTR		Percentage Correct
			No	YES	
Step 0	ACCEPTCONTR	No	0	190	,0
		YES	0	594	100,0
	Overall Percentage				75,8

a. Constant is included in the model.
b. The cut value is ,500

Variables in the Equation

		B	S.E.	Wald	df	Sig.	Exp(B)
Step 0	Constant	1,140	,083	187,035	1	,000	3,126

Variables not in the Equation

			Score	df	Sig.
Step 0	Variables	AGE5	12,773	4	,012
		AGE5(1)	7,283	1	,007
		AGE5(2)	2,408	1	,121
		AGE5(3)	4,538	1	,033
		AGE5(4)	1,344	1	,246
		Status2(1)	5,558	1	,018
		Preg3	8,984	2	,011
		Preg3(1)	1,449	1	,229
		Preg3(2)	2,604	1	,107
		Q3.9(1)	4,364	1	,037
	Overall Statistics		22,859	8	,004

Block 1: Method = Enter

Omnibus Tests of Model Coefficients

		Chi-square	df	Sig.
Step 1	Step	22,783	8	,004
	Block	22,783	8	,004
	Model	22,783	8	,004

Model Summary

Step	-2 Log likelihood	Cox & Snell R Square	Nagelkerke R Square
1	845,529[a]	,029	,043

a. Estimation terminated at iteration number 4 because parameter estimates changed by less than ,001.

Hosmer and Lemeshow Test

Step	Chi-square	df	Sig.
1	6,006	8	,647

Contingency Table for Hosmer and Lemeshow Test

		ACCEPTCONTR = No		ACCEPTCONTR = YES		
		Observed	Expected	Observed	Expected	Total
Step 1	1	28	28,863	46	45,137	74
	2	27	22,061	41	45,939	68
	3	24	22,095	49	50,905	73
	4	16	20,689	59	54,311	75
	5	17	19,323	61	58,677	78
	6	17	18,159	61	59,841	78
	7	21	16,769	61	65,231	82
	8	12	11,473	51	51,527	63
	9	9	11,757	59	56,243	68
	10	19	18,812	106	106,188	125

Classification Table[a]

			Predicted		
			ACCEPTCONTR		Percentage Correct
	Observed		No	YES	
Step 1	ACCEPTCONTR	No	0	190	,0
		YES	0	594	100,0
	Overall Percentage				75,8

a. The cut value is ,500

Variables in the Equation

		B	S.E.	Wald	df	Sig.	Exp(B)	95% C.I.for EXP(B)	
								Lower	Upper
Step 1[a]	AGE5			10,615	4	,031			
	AGE5(1)	,455	,420	1,172	1	,279	1,576	,692	3,590
	AGE5(2)	,853	,391	4,757	1	,029	2,347	1,090	5,054
	AGE5(3)	,798	,385	4,294	1	,038	2,220	1,044	4,722
	AGE5(4)	,132	,414	,102	1	,749	1,141	,507	2,568
	Status2(1)	,145	,216	,450	1	,503	1,156	,757	1,764
	Preg3			6,032	2	,049			
	Preg3(1)	,394	,211	3,508	1	,061	1,483	,982	2,241
	Preg3(2)	,658	,279	5,568	1	,018	1,932	1,118	3,337
	Q3.9(1)	,192	,184	1,083	1	,298	1,211	,844	1,739
	Constant	-,019	,426	,002	1	,965	,981		

a. Variable(s) entered on step 1: AGE5, Status2, Preg3, Q3.9.

Correlation Matrix

		Constant	AGE5(1)	AGE5(2)	AGE5(3)	AGE5(4)	Status2(1)	Preg3(1)	Preg3(2)	Q3.9(1)
Step 1	Constant	1,000	-,890	-,865	-,757	-,650	-,289	-,300	-,480	-,035
	AGE5(1)	-,890	1,000	,845	,768	,653	,216	,140	,396	-,075
	AGE5(2)	-,865	,845	1,000	,805	,690	,132	,084	,359	-,082
	AGE5(3)	-,757	,768	,805	1,000	,682	,069	-,003	,214	-,090
	AGE5(4)	-,650	,653	,690	,682	1,000	,044	,002	,090	-,020
	Status2(1)	-,289	,216	,132	,069	,044	1,000	-,172	-,215	-,363
	Preg3(1)	-,300	,140	,084	-,003	,002	-,172	1,000	,553	-,010
	Preg3(2)	-,480	,396	,359	,214	,090	-,215	,553	1,000	,017
	Q3.9(1)	-,035	-,075	-,082	-,090	-,020	-,363	-,010	,017	1,000
